# Long-Term Remission Rate of Type 2 Diabetes Following Bariatric Surgery: A Retrospective Cohort Study in Adult Patients in Riyadh, Saudi Arabia

**DOI:** 10.7759/cureus.76819

**Published:** 2025-01-02

**Authors:** Bader Altulaihi, Ali M Sawlan, Nemer A Alwahbi, Bandar Alshahrani, Yazeed H Alrayani, Yazan H Alrayani

**Affiliations:** 1 Department of Family and Community Medicine, King Abdullah International Medical Research Center, Ministry of National Guard - Health Affairs, Riyadh, SAU; 2 Department of Family Medicine, King Abdulaziz Medical City, Riyadh, SAU; 3 College of Medicine, King Saud Bin Abdulaziz University for Health Sciences College of Medicine, Riyadh, SAU; 4 College of Medicine, King Saud Bin Abdulaziz University for Health Sciences, Riyadh, SAU

**Keywords:** bariatric surgery and diabetes, diabetes mellitus type 2, kingdom of saudi arabia (ksa), long-term outcome, type 2 diabetes remission

## Abstract

Introduction

Type 2 Diabetes Mellitus (T2DM) is a chronic metabolic disorder that poses significant health and economic burdens, particularly in regions like Saudi Arabia where prevalence rates are among the highest globally. Bariatric surgery has emerged as a promising intervention, not only for weight reduction but also for inducing diabetes remission. However, while the short-term benefits of the procedure are well documented, long-term outcomes remain under-explored, especially in Saudi populations.

This study aims to evaluate the long-term remission rates of T2DM and weight measurements following bariatric surgery in adult Saudi patients in Riyadh.

Methods

Using a retrospective cohort design, this study analyzes medical records of patients aged 18-65 who underwent bariatric surgery in the period of 2016-2020 and assesses Hemoglobin A1c (HbA1c) levels pre and post-surgery and diabetes remission using American Diabetes Association (ADA) criteria. The research also investigates factors influencing relapse rates and potential gender differences in remission outcomes.

Results

Out of 74 participants, the majority were females (53, 71.62%) compared to males (21, 28.37%). The mean BMI was recorded at 44.91 ± 6.68 kg/m^2^, indicating that participants generally fell within the obesity weight range. The mean HbA1c level prior to surgery was 8.70 ± 1.68, indicating suboptimal glycemic control at baseline. The mean HbA1c level decreased from 8.70 ± 1.68 before surgery to 6.76 ± 1.42 at the 12-month mark. The results indicated significant improvements in glycemic control post-surgery, as evidenced by the marked reduction in HbA1c levels at various time points (1 year, 2 years, etc.) with a p-value of 0.000. In terms of remission, the data showed a distinct result: out of 74 participants, only 20 participants (27%) achieved diabetes remission. Furthermore, seven participants (35%) out of the diabetic remission group experienced a relapse post-intervention and one participant achieved a second diabetes remission. In the assessment of risk factors for relapse, baseline BMI and gender were not strong factors in determining whether a patient experiences diabetes relapse post-surgery.

As for weight measurements, the findings showed a steady increase in both Percent Excess Weight Loss (%EWL) and Percent Total Weight Loss (%TWL) in the initial years following surgery with no significant variations across the years, and the p-values were 0.710 and 0.446, respectively. For Percent Weight Regain (%WR), there was a significant and steady increase across the period, with values starting at 6.77 ± 11.8% at Year 2, increasing over the years, and peaking again at 26.64 ± 44.84% by Year 5, with a p-value of < 0.001.

Conclusion

This study is consistent with previous studies and provides compelling evidence that bariatric surgery leads to substantial improvements in glycemic control and weight reduction among Saudi patients with T2DM. However, it shows a lower percentage of remission in our participants compared to other local and global studies. Further local research is recommended to confirm our findings, analyze the reason for lower rates, and investigate the predictors of remission and relapse. This study fills a crucial gap in localized data, providing insights that could guide future healthcare strategies and improve the management of T2DM in the region.

## Introduction

Diabetes mellitus type 2 (T2DM), which represents around 90% of diabetes, is a long-term, chronic, persistent metabolic disorder characterized by hyperglycemia due to the body’s inability to regulate blood sugar levels of insulin. Chronic high blood glucose levels are associated with long-term damage, dysfunction, and failure of different organs. T2DM is a multifactorial disease and some of the risk factors include genetic predisposition, aging, and obesity, which increases the risk of diabetes by 80-100 folds. The epidemiology is largely variable around the world, although the highest rates are in the Middle East and Pacific Islands [1].

In the Gulf Cooperation Council (GCC) countries, there has been a growing trend of T2DM. One of the reasons that can explain the growing trend is that Arabs are genetically susceptible to developing the disease [2]. In Saudi Arabia, the prevalence of diabetes has increased approximately ten-fold in three decades from 1982 to 2004 [2-6]. Two large studies were done to assess the prevalence of diabetes in Saudi Arabia. The first one was published in 2004 and included 17,232 Saudi participants with a 98.2% response rate revealing that 4004, 23.7%, subjects were diabetic [7]. The other one was done from 2007 to 2009 and involved 18,034 participants, aged 30 years old or older. It found that 25.4% were pre-diabetic and 25.5% were diagnosed with diabetes mellitus (DM) [8]. This puts a significant economic burden on the government. The Saudi Ministry of Health spent in 2010 $0.87 billion dollars to treat diabetes, and the cost was expected to be $6.5 billion dollars by 2020 [9].

The management of T2DM primarily focuses on lifestyle modification and weight reduction, which plays a major role in insulin resistance and the development of T2DM; medications are added when needed to achieve the target glycemic control. Another modality of treatment is bariatric/metabolic surgery, which underwent a significant improvement and developed many procedures that resulted in success in reducing weight and T2DM remission in the short term (1-2 years) [10]. However, in the long-term results, many patients experienced T2DM relapse and weight regain. For example, in the Swedish Obese Subjects (SOS) trial, 50% of patients who achieved DM remission at 2 years relapsed after 10 years [11]. In addition, a retrospective study done on patients who underwent the surgery between 2008 and 2011 revealed that half of the subjects regained weight after 7 years [12]. A local study done in King Saud University Hospital revealed that 53.3% regained 25% or more of their lowest weight after 6 years of the surgery [13]. Another recent study, which is the only one of its kind in Saudi Arabia, done to assess diabetes remission in Saudi patients following bariatric surgery found that 48.5% achieved diabetes remission according to the American Diabetes Association criteria that was published in 2019 [14]. However, the study included participants who underwent the surgery 1 year ago, which could have an influence on the results.

In conclusion, bariatric surgery could be a solution for the high T2DM rates and the obesity epidemic, which is a major risk factor for developing the disease in Saudi Arabia, and that will result in a healthier population and lower expenditure. However, local data regarding the impact of the surgery on T2DM remission and maintenance of healthy body weight in the long term are lacking. Such research is needed since there are genetic and diet differences between countries. The aim of the study is to assess the T2DM remission rate in the long term following bariatric surgery in Saudi patients.

## Materials and methods

Ethical and study approval

This is a retrospective cohort study that was conducted in King Abdulaziz Medical City, Riyadh, Saudi Arabia. It was approved by the Institutional Review Board (IRB) of King Abdullah International Medical Research Center (KAIMRC) (IRB No. IRB/2477/23), approved on 10 October 2023 and the study duration was 1 year.

Patient data and inclusion criteria

The study included all patients who were 18-65 years old, diagnosed with T2DM, and patients who underwent bariatric surgery for weight reduction at least 3 years ago. Patients were excluded if they were i) younger than 18 or older than 65 years, ii) not diagnosed with diabetes or diagnosed with diabetes other than T2DM, or iii) underwent bariatric surgery less than 3 years ago. The patients were identified and collected from the existing Electronic Medical Records in our hospital, and the collected data included date of birth, gender, height, weight before surgery and on each physical visit, BMI, date of diagnosis of T2DM, type of surgery, hemoglobin A1c (HbA1c) levels, and the use of hypoglycemic agents.

Diabetes remission criteria

The diabetes remission criteria used in this study were the American Diabetes Association (ADA) criteria [15]. Diabetes remission was defined as an HbA1c level of <6.5 for at least three months without administering any hypoglycemic medication. Additional considerations in the ADA criteria were: i) if HbA1c was discovered to be unreliable, fasting blood glucose would be the alternative, ii) HbA1c should be performed prior to the intervention and repeated no sooner than three months, and iii) subsequent testing to assess the longevity of the remission favorable to be done at least yearly. To align these criteria with our study and hospital follow-up, complicated cases, such as malignancy and various hematological diseases, that may affect HbA1c reliability were excluded, three readings for HbA1c were obtained before the surgery, and all readings were obtained post-surgery.

Weight calculation formulas

For weight measurements, we calculated %Excess weight loss (%EWL) as follows:

$\%EWL = \left( \frac{\text{pre-surgery weight} - \text{follow-up weight}}{\text{operative excess weight}} \right) \times 100$

The %Total Weight Loss (%TWL) was calculated using the following formula:

$\%TWL = \left( \frac{\text{preceding year weight} - \text{current weight}}{\text{preceding year weight}} \right) \times 100$

The %Weight Regain (%WR) is defined as the weight regained after reaching the lowest weight, and it was calculated as follows:

$\%WR = \left( \frac{\text{current weight} - \text{nadir weight}}{\text{pre-surgery weight} - \text{nadir weight}} \right) \times 100$

Sample size

**Figure 1:**
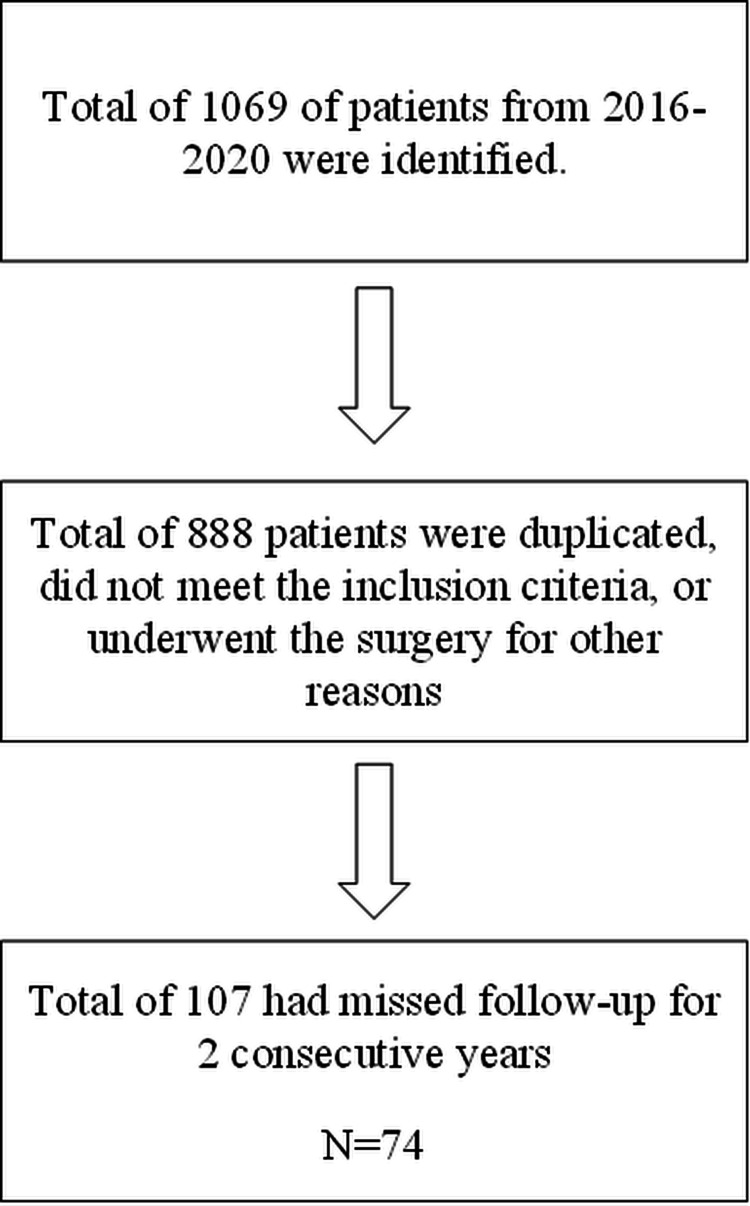
Flow chart of sample size selection

There were 2035 patients who underwent bariatric surgery in KAMC from 2016 until the date of data request in 2023. 1069 patients underwent the surgery in 2016-2020. 888 patients either were duplicated, excluded for not meeting the criteria, or underwent the surgery for other purposes. Lack of follow-up was noted, so an additional criterion was added, and any patient who missed follow-up for consecutive 2 years was excluded, and the remaining sample was 74. The flow chart of sample size selection is shown in Figure 1.

Statistical analysis

The SPSS for Windows version 22 was used (IBM Corp., Armonk, USA). A repeated-measures design was utilized to evaluate the longitudinal impact of bariatric surgery on glycemic control and weight management among participants over five years and included some adjustments for missing data. The study tracked changes in HbA1c levels, percentage excess weight loss (%EWL), total weight loss (%TWL), and weight regain (%WR) at annual intervals post-surgery. Gender-specific comparisons were also conducted to examine potential variations in outcomes between male and female participants. Statistical analyses included ANOVA for repeated measures to assess temporal trends and interactions, alongside logistic regression to identify predictors of diabetic remission and relapse. Significance thresholds were set at p < 0.05 with a confidence interval of 95%. Data collection encompassed demographic and clinical variables, with baseline and follow-up measures standardized for consistency. This robust design allowed for the analysis of both individual and group-level patterns in post-surgical outcomes.

## Results

Out of 74 participants, the majority were female (53, 71.62%) compared to males (21, 28.37%). The mean height of participants was 159.8 cm with a standard deviation of 9.07 cm, while the mean weight was 114.79 kg (± 19.18 kg). The mean BMI was recorded at 44.91 ± 6.68 kg/m^2^, indicating that participants generally fell within the obesity weight range.

In terms of diabetic remission, 20 participants (27%) out of the 74 patients achieved diabetes remission. Furthermore, seven participants (35%) out of the diabetic remission group experienced a relapse post-intervention and one participant (14%) achieved a second diabetes remission. The mean HbA1c level prior to surgery was 8.70 (± 1.68), indicating suboptimal glycemic control at baseline. The average estimated weight loss (%EWL) was 60.25% (± 21.48%), while the average total weight loss (%TWL) was 30.04% (± 8.90%), suggesting significant weight loss following bariatric surgery. Lastly, the average weight regain was found to be 14.26 ± 26.39% as shown in Table 1.

**Table 1:** Summary of the demographic characteristics and clinical measurements of the study participants. Summary of the demographic characteristics and clinical measurements of the study participants, including gender distribution, height, weight, body mass index (BMI), diabetic remission (DR) status, relapse status, second diabetic remission (DR 2), second relapse status (Relapse 2), hemoglobin A1C Before (HbA1cB) levels, and weight loss metrics (Percent Excess Weight Loss (%EWL), Percent Total Weight Loss (%TWL), Percent Weight Regain (%WR)).

Category		N (%)	Minimum/Maximum	Mean ± SD
Gender	Male	21 (28.37)	-	-
	Female	53 (71.62)	-	-
Height (cm)	-	-	150-190	159.8 ± 9.07
Weight (kg)	-	-	77-180.4	114.79 ± 19.18
BMI (kg/m^2^)	-	-	32.5-77.1	44.91 ± 6.68
DR	Yes	20 (27)	-	-
Relapse	Yes	7 (35)	-	-
DR 2	Yes	1 (14)	-	-
Relapse 2	No	0 (0)	-	-
HbA1cB	-	-	6.1-12.5	8.70 ± 1.68
%EWL	-	-	14.5-115.3	60.25 ± 21.48
%TWL	-	-	3.9-55.3	30.04 ± 8.90
%WR	-	-	-	14.26 ± 26.39

The pre-surgery HbA1c level (HbA1cB) was measured at 8.70 (± 1.68). At the one-year mark post-surgery (HbA1c12M), the mean HbA1c level significantly decreased to 6.76 (± 1.42), with an F-value of 52.85 and a p-value of 0.00, indicating a highly significant change in HbA1c levels. Subsequent measurements, as summarized in Table 2, at 2 years (HbA1c24M), 3 years (HbA1c36M), 4 years (HbA1c48M), and 5 years (HbA1c60M) showed HbA1c levels of 6.71 (± 1.27), 6.81 (± 1.32), 6.78 (± 1.27), and 6.82 (± 1.25), respectively, although no additional F-values or p-values are provided for these later time points.

**Table 2:** Summary of the mean HbA1c levels of participants at various time points before and after surgery. ANOVA is the statistical test used to analyze the p-value.

Time Point	Mean ± SD	F-value	p-value
HbA1c Before Surgery (HbA1cB)	8.70 ± 1.68		
HbA1c at 1 Year (HbA1c12M)	6.76 ± 1.42	52.85	.00
HbA1c at 2 Years (HbA1c24M)	6.71 ± 1.27		
HbA1c at 3 Years (HbA1c36Madjusted)	6.81 ± 1.32		
HbA1c at 4 Years (HbA1c48Madjusted)	6.78 ± 1.27		
HbA1c at 5 Years (HbA1C60Madjusted)	6.82 ± 1.25		

Table 3 summarizes the significant pairwise comparisons of HbA1c levels across different time points, highlighting the mean differences and associated p-values. The comparisons demonstrate a statistically significant reduction in HbA1c levels when comparing the pre-surgery measurement (HbA1cB) to those at the 1-year (HbA1c12M), 2-year (HbA1c24M), 3-year (HbA1c36M), 4-year (HbA1c48M), and 5-year (HbA1c60M) marks, with all p-values reported as 0.000. The mean differences ranged from 1.88 to 1.99, indicating substantial improvements in glycemic control over time. In contrast, there were no significant differences noted between subsequent years (e.g., HbA1c12M vs. HbA1c24M), with p-values all at 1.00, indicating stability in HbA1c levels after the initial postoperative year.

**Table 3:** Summary of significant pairwise comparisons of HbA1c levels

Comparison	Mean Difference	p-value
HbA1cB (Before Surgery) vs. HbA1c12M (1 Year)	1.94	.000
HbA1cB vs. HbA1c24M (2 Years)	1.99	.000
HbA1cB vs. HbA1c36M (3 Years)	1.89	.000
HbA1cB vs. HbA1c48M (4 Years)	1.92	.000
HbA1cB vs. HbA1c60M (5 Years)	1.88	.000
HbA1c12M vs. HbA1c24M	0.05	1.00
HbA1c12M vs. HbA1c36M	-0.04	1.00
HbA1c12M vs. HbA1c48M	-0.01	1.00
HbA1c12M vs. HbA1c60M	-0.05	1.00
HbA1c24M vs. HbA1c36M	-0.09	1.00
HbA1c24M vs. HbA1c48M	0.02	1.00
HbA1c24M vs. HbA1c60M	-0.11	1.00
HbA1c36M vs. HbA1c48M	0.01	1.00
HbA1c36M vs. HbA1c60M	0.04	1.00
HbA1c48M vs. HbA1c60M	-0.04	1.00

The odds ratio (OR) for gender is 2.16, with a 95% confidence interval (CI) of (0.10, 45.05) and a p-value of 0.49. This result indicates that while the odds of diabetic remission are higher for males compared to females, the association is not statistically significant. The analysis showed that baseline BMI does not significantly indicate relapse after bariatric surgery in diabetic patients. The odds ratio of 1.02 suggests a minimal 2.5% increase in the odds of relapse for each unit increase in BMI, but this effect is not statistically significant since the p-value is 0.65 (Table 4). These findings suggest that baseline BMI is not a strong factor in determining whether a patient experiences diabetes relapse post-surgery, and other variables may play a more significant role.

**Table 4:** The results of a logistic regression analysis to identify the predictors of relapse of type 2 diabetes mellitus following bariatric surgery. Logistic regression is the statistical used to analyze the p-value

Variable	OR (Exp(B))	p-Value	95% CI for OR
Gender (Male and Female)	2.16	0.49	(0.10, 45.05)
BMI (kg/m^2^)	1.02	0.65	(0.92, 1.14)

The mean %EWL values for the first five years were recorded as follows: 58.91% (± 29.67%), 61.55% (± 25.79%), 60.77% (± 26.47%), 62.73% (± 27.18%), and 57.27% (± 43.82%). The F-value for the %EWL across time points was 0.48, with a corresponding p-value of 0.71, indicating no statistically significant difference in %EWL over the observed years.

For %TWL, the values were 28.44% (± 8.25%), 30.25% (± 9.03%), 29.82% (± 9.80%), 30.79% (± 10.51%), and 30.90% (± 18.83%). The F-value for %TWL was 0.93 with a p-value of 0.44, suggesting a lack of significant variation in %TWL over the five-year period.

In contrast, %WR showed a statistically significant change across the time points, with values starting at 6.77 ± 11.8% at Year 2, increasing over the years, and peaking again at 26.64 ± 44.84% by Year 5. The F-value for %WR was 239.96 with a p-value of < 0.001, indicating a significant overall difference across years for %WR. Weight measurements are shown in Table 5.

**Table 5:** The mean percentage of excess weight loss (%EWL), percent total weight loss (%TWL), and percent weight regain (%WR) at different time points post-surgery over five years. ANOVA is the statistical test used to analyze the p-value

Time Points (in Years)	1st	2nd	3rd	4th	5th	F-Value	p-Value
%EWL	58.91 ± 29.67	61.55 ± 25.79	60.77 ± 26.47	62.73 ± 27.18	57.27 ± 43.82	0.53	0.71
%TWL	28.44 ± 8.25	30.25 ± 9.03	29.82 ± 9.80	30.79 ± 10.51	30.90 ± 18.83	0.93	0.44
%WR		6.77 ± 11.8	11.06 ± 15.17	12.55 ± 15.02	26.64 ± 44.84	239.96	< 0.001

## Discussion

The results of the study showed significant improvements in DM and sustained lower HbA1c levels post-surgery, as evidenced by the marked reduction in HbA1c levels at various time points (1 year, 2 years, etc.). The gender distribution in our cohort, with females comprising 71.62% and males 28.37%, aligns with broader trends observed in bariatric surgery populations worldwide. This consistency suggests that gender-specific factors, such as higher referral rates and patient-driven requests due to body image concerns, may play a significant role in the decision to pursue bariatric surgery, highlighting the need for targeted interventions to address potential disparities in access or healthcare-seeking behaviors [16]. 

The most pronounced finding of this study is the markedly lower rates of DM remission following bariatric surgery compared to both local and global literature. While the average remission rate reported in previous global studies hovers around 50%, a local study reported a remission rate of 48.5% [14,17-19]. Our cohort demonstrated a substantially reduced rate of only 27%. This discrepancy is not only statistically significant but also clinically relevant, warranting careful examination and interpretation.

Several factors may contribute to this notable disparity. Firstly, with regard to patient demographics and preoperative characteristics, our study population may differ in key aspects such as age, duration of diabetes, severity of insulin resistance, or preoperative BMI. These factors might have an influence on surgical outcomes and could partially explain the lower remission rates observed. Secondly, some clinicians may still prescribe antidiabetic medication after the surgery even though the medication could be ceased. This could be a major cause and result in lower participants who achieved DR depending on ADA criteria. Another possible factor is the lower sample size than anticipated, which is caused by the lack of follow-up (for two consecutive years) that led to many participants being expelled from the cohort study. In this specific aspect, our research appears to have more stringent criteria compared to other research. Another considerable factor is the variation in the criteria across published papers.

The substantial difference in remission rates emphasizes the need for population-specific research and tailored approaches to bariatric surgery. It challenges the generalizability of global averages to all populations and highlights the importance of managing patient expectations based on local data. This study may influence local clinical practice by emphasizing the necessity of follow-up after surgery. 

Limitations

Firstly, a significant challenge encountered during data collection for our study on the rate of diabetic remission following bariatric surgery was the substantial loss of follow-up among participants. This attrition resulted in missing participants, which poses a considerable limitation in accurately assessing long-term outcomes. This might have an impact on the results of diabetes remission in this study. Some patients who lost follow-up may have developed diabetes remission afterward and vice versa. The discontinuation of follow-up care is not uncommon in bariatric surgery cohorts, as highlighted by previous studies indicating a marked decline in patient compliance over time. For instance, follow-up rates can drop drastically from 90% in the first year to less than 10% after 10 years [20]. This trend underscores the necessity for strategies to enhance patient retention and ensure comprehensive data collection, thereby enabling more robust and reliable conclusions regarding the efficacy of bariatric surgery in achieving sustained diabetes remission.

Secondly, the substantial loss to follow-up not only resulted in missing participants but also significantly reduced the number of patients eligible for inclusion in our study. This reduction in sample size poses a considerable challenge to the statistical power and generalizability of our findings. The stringent inclusion criteria, coupled with the high attrition rate, led to a smaller cohort than initially anticipated. Consequently, our analysis may be limited in its ability to capture the full spectrum of outcomes across diverse patient profiles. This underscores the critical need for innovative retention strategies and perhaps the implementation of multiple imputation techniques or sensitivity analyses to mitigate the impact of missing data on the validity of our conclusions.

Thirdly, the relatively new Electronic Medical Records did not include patients who followed up before the year of the introduction of the system.

## Conclusions

This study is consistent with previous studies and fills a crucial gap in localized data, providing insights that could guide future healthcare strategies and improve the management of T2DM in the region. It provides compelling evidence that bariatric surgery leads to substantial improvements in glycemic control and weight reduction among Saudi patients with T2DM. However, it shows a lower percentage of remission in our participants compared to other local and global studies and raises an important concern for follow-up of the patients. Further local research is recommended to confirm our findings, analyze the reason for lower rates, and investigate the predictors of remission and relapse.
